# Cost savings of reducing opioid prescribing for the treatment of people with low back pain in general practice: a modelling study

**DOI:** 10.1016/j.lanwpc.2024.101277

**Published:** 2025-01-10

**Authors:** Anagha Killedar, Romi Haas, Alexandra Gorelik, Sean Docking, Rachelle Buchbinder, Chris G. Maher, Chung-Wei Christine Lin, Alison Hayes

**Affiliations:** a Leeder Centre for Health Policy, Economics and Data, School of Public Health, Faculty of Medicine and Health, University of Sydney, NSW, 2006, Australia; b School of Public Health, Faculty of Medicine and Health, University of Sydney, NSW, 2006, Australia; c Musculoskeletal Health and Wiser Health Care Units, School of Public Health and Preventive Medicine, Monash University, Melbourne, VIC, 3004, Australia; d Institute for Musculoskeletal Health, Sydney Local Health District, Sydney, NSW, Australia

**Keywords:** Opioids, Low back pain, Healthcare savings, Cost savings, General practice, Economic modelling

## Abstract

**Background:**

Low back pain (LBP) is the leading cause of disability worldwide. Contrary to clinical guidelines, opioids are frequently prescribed early in the management of LBP in primary care, leading to potential harm and downstream healthcare costs. The objective of this study was to model the one-year impacts of strategies that reduce opioid prescribing for low back pain (LBP) in primary care on healthcare costs and overdose deaths Australia-wide and explore the potential for such strategies to be cost-neutral.

**Methods:**

Two decision tree models were developed: the healthcare actions model, which tracked post-diagnosis care pathways, and the opioid overdose model, which modelled overdoses and consequent healthcare costs and deaths, following opioid prescribing. These models were developed using data from the electronic medical records of 65,612 LBP patients from general practices in Victoria, Australia and from published literature. Healthcare costs and change in overdose deaths associated with strategies delivering 0–100% relative reduction in opioid prescribing for LBP in primary care were estimated with a one-year time horizon. The relative reduction in opioid prescription needed for a strategy to be cost-neutral was also calculated.

**Findings:**

A relative 20% reduction in opioid prescribing was estimated to save $5.41 million due to changes to downstream care, save $2.24 million due to avoided opioid overdoses and prevent 81 overdose deaths nationally, over one year. A relative reduction in opioid prescribing of 1.2% and 10.3% would be needed to recoup the costs of a strategy costing $500,000 and $4 million, respectively, over one year.

**Interpretation:**

The study highlights the short-term health and economic benefits of reducing opioid prescribing for LBP and suggests that a low to medium intensity strategy could be cost-neutral or cost-saving.

**Funding:**

This study was funded by the 10.13039/501100000925National Health and Medical Research Council of Australia.

**Research in context**

**Evidence before this study**

Contrary to clinical recommendations, patients with low back pain (LBP) visiting a general practitioner (GP) are frequently prescribed opioids at an early stage of treatment. To examine the literature on changes to GP opioid prescribing practices for LBP and impacts on healthcare costs, we searched Medline using a combination of MeSH terms (“Analgesics, Opioid”, “Low Back Pain”, “General Practice”, “General Practitioners”, “Family Practice”, “Primary Health Care”, “Health Care Costs” and “Cost Savings”) and keyword searches (“opioid”, “low back pain”, “general practice”, “general practitioner”, “primary care”, “healthcare cost”). Dates were from inception to 2023. The search returned six hits. In all six studies, the type of healthcare provider, not opioid prescriptions, was the main exposure examined in the study, with a large focus on physical therapy. Opioid prescriptions or use was an outcome in five of the studies and one study found that opioids was a predictor of healthcare costs.

**Added value of this study**

This is the first study, internationally, to quantify the potential healthcare cost savings from reducing opioid prescribing for low back pain in the general practice setting. We demonstrate that the savings are likely to recoup the costs of a moderately priced intervention, even with a modest effect on opioid prescribing. We additionally estimate the number of lives saved from avoided opioid overdoses by reducing opioid prescribing.

**Implications of all the available evidence**

The evidence indicates the strong potential for strategies to reduce opioid prescribing to be net cost-saving and reduce overdose mortality, subject to the effectiveness of the strategy. This highlights the need to identify and trial strategies that are effective at reducing opioid prescriptions for low back pain.

## Introduction

Low back pain (LBP) is the leading cause of disability worldwide, with the most recent Global Burden of Disease study estimating its global point prevalence at 7.5%.^1^ In Australia, 16% of the population reported experiencing back pain in 2022.^2^ When treating LBP, the recently published Australian LBP Clinical Care Standard^3^ recommends that the majority of patients should initially be offered education and advice, encouraged to self-manage and engage in physical activity. These actions should be attempted before resorting to the prescription of pain medications such as opioids, diagnostic imaging, and referrals for specialist review.^3^

Despite these standards, evidence-based care for people with LBP is underutilised in the general practice setting.^4^, ^5^, ^6^ Globally and in Australia, only 20% of patients visiting a general practitioner are provided evidence-based information and advice.^5^^,^^7^ Additionally, observational evidence from Australia has demonstrated that 36% of patients are prescribed an opioid within a year of being diagnosed with low back pain^8^ which is consistent with international evidence.^5^ Early opioid prescriptions for people with LBP may initiate downstream care that may not improve health outcomes^9^ and even cause harm. For example, opioid treatment has been shown to increase the risk of surgery and ongoing opioid use,^10^ and establish a path to related harms such as opioid overdose and abuse.^11^ Opioid treatment has also been found to be a predictor of healthcare costs.^12^

In 2018, the Lancet published a call for action on LBP, with a particular focus on preventing the over-medicalisation of LBP and reducing inappropriate care.^13^ In line with this call, strategies that aim to reduce opioid prescribing have been attempted in recent years including research trials^14^ and the release of clinical guidelines and clinical care standards,^3^^,^^15^ with varying or unexamined effects. However, shifting prescribing rates at a national level is dependent on large-scale behaviour change, an endeavour that will likely require commitment to effective strategies by various players including government, medical practices, the practitioner training sector, general practitioners (GPs), and patients with LBP themselves. To incentivise such commitment in each of these players, a comprehensive understanding of the health and economic benefits of reducing prescriptions of opioids is necessary. While there is growing evidence that opioid prescriptions are not effective in treating acute LBP^9^ and increase the risk of harms,^11^ the extent to which strategies to reduce opioid prescribing could reduce healthcare costs and mortality are not known. Therefore, the aim of this study was to model the changes to care pathways and potential impacts on healthcare costs and overdose deaths at a population level from strategies reducing opioid prescribing for LBP in general practice. A second aim was to explore the potential for the costs of such strategies to be recouped in downstream healthcare savings and determine the effectiveness of a strategy required to achieve this.

## Methods

Two decision analytic models of LBP care pathways were developed to investigate healthcare cost savings associated with reducing opioid prescribing by GPs. The models incorporate the deprescribing of opioids for those currently prescribed opioids and the reductions of first-time prescribing in opioid naïve patients. The first model followed healthcare actions subsequent to an LBP diagnosis by a GP and the second model captured downstream impacts on opioid overdoses. Analyses conducted from each model took a healthcare perspective, capturing costs to Australian health payers including the government and private health funders. Costs were valued in 2022 Australian Dollars, using the Australian health price index^16^ to inflate values as necessary. The time horizon of all analyses was one year after diagnosis of LBP. No discounting was applied as outcomes beyond one year were not included. All analyses were conducted in Treeage Pro Healthcare 2023. The two models are described below.

### Healthcare actions model

The ‘healthcare actions model’ was developed using the POpulation Level Analysis and Reporting (POLAR) database as the key data source.^17^ The extract of data from this database used in this analysis included de-identified patient-related data from all electronic medical records of the 301 consenting practices within the three Primary Health Networks (PHNs) of Eastern Melbourne, South-Eastern Melbourne and Gippsland within Victoria, Australia.^18^ The population captured by these networks is ethnically and socioeconomically diverse,^19^ reflecting the Australian population of which 26% are born overseas, 4% are indigenous, and 37% of adults only have school-level qualification and 33% are not in the labour force.^20^

The cohort in our study included all patients aged ≥18 years with at least one GP face-to-face consultation and an eligible low back diagnostic code between 1st January 2014 and 31st December 2018 (n = 65,612). Patients with an LBP diagnosis were identified using SNOMED CT-AU terminology as previously described.^17^ We excluded traumatic diagnoses and inflammatory and autoimmune rheumatic diseases. As determined previously,^18^ 53% of the included cohort were women, mean age was 49.2 (SD 18.5) years, 87% lived in a metropolitan location, and 62% had a least one chronic comorbidity (most commonly cardiovascular, anxiety/depression, or musculoskeletal). For each included patient, dates and types of GP consultations, prescriptions for pain relief, requests for diagnostic imaging, and referrals to other healthcare practitioners were quantified after the first recorded eligible diagnosis of low back pain.

**Fig. 1:**
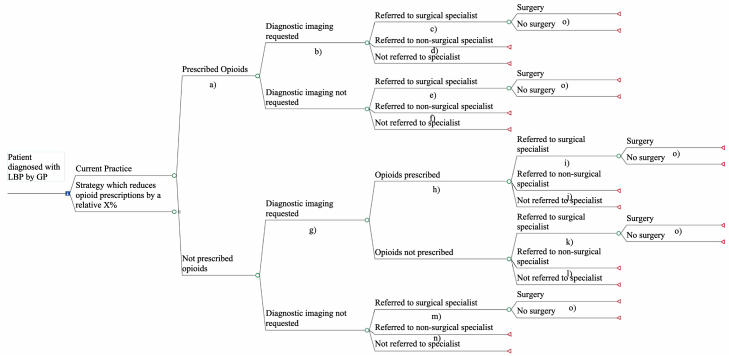
Schematic of healthcare actions decision tree model.

Our model (Fig. 1) examined a hypothetical decision to implement a policy or intervention that reduces opioid prescribing by a specified amount. This is represented in the decision node (square node). A decision tree model was developed that mapped the likely sequence of GP actions after the index event (i.e. diagnosis of LBP) with the first chance node (circle node) indicating whether opioids were prescribed as a first action or not. The actions after this were selected to follow the most common sequence of GP actions after an LBP diagnosis as identified from the POLAR database and allowed for opioid prescriptions at two points (before and after an imaging request).

The conditional probabilities of most actions were derived from the cohort of patients in the POLAR database described above (Table 1). Actions within the one year period after diagnosis of low back pain were counted, except for those diagnosed in 2018 for whom less than one year of follow-up data were available. These were complemented by data from published literature on surgery in low back pain patients referred to orthopaedic specialists^21^ as the POLAR database does not include records of healthcare actions outside of general practice.

**Table 1:** Parameters for healthcare actions model.

Label	Model parameter	Base estimate	Distribution	Data source
	Probabilities			

a)	Probability of being prescribed opioids as first action under current practice	0.329	*Beta (n* = 65,612, *r* = 21,603*)*	POLAR database
b)	Probability of having imaging requested if prescribed opioids	0.169	*Beta (n* = 21,603*, r* = 3655*)*	POLAR database
c)	Probability of surgical referral if prescribed opioids and imaging requested	0.157	*Beta (n* = 3655, *r* = 575*)*	POLAR database
d)	Probability of non-surgical referral if prescribed opioids and imaging requested	0.063	*Beta (n* = 3655, *r* = 231*)*	POLAR database
e)	Probability of surgical referral if prescribed opioids and no imaging requested	0.083	*Beta (n* = 17,948, *r* = 1492*)*	POLAR database
f)	Probability of non-surgical referral if prescribed opioids and no imaging requested	0.060	*Beta (n* = 17,948, *r* = 1084*)*	POLAR database
g)	Probability of having imaging requested if not prescribed opioids as first action	0.167	*Beta (n* = 44,009 *r* = 7333*)*	POLAR database
h)	Probability of having opioids prescribed if not prescribed opioids first, and imaging requested	0.175	*Beta (n* = 7333, *r* = 1280*)*	POLAR database
i)	Probability of surgical referral if requested imaging first, and subsequently opioids	0.155	*Beta (n* = 1280, *r* = 198*)*	POLAR database
j)	Probability of non-surgical referral if requested imaging first, and subsequently opioids	0.062	*Beta (n* = 1280, *r* = 79*)*	POLAR database
k)	Probability of surgical referral if requested imaging first, and no opioids	0.113	*Beta (n* = 6053, *r* = 683*)*	POLAR database
l)	Probability of non-surgical referral if requested imaging first, and no opioids	0.054	*Beta (n* = 6053, *r* = 329*)*	POLAR database
m)	Probability of surgical referral if NOT prescribed opioids or requested imaging first	0.063	*Beta (n* = 36,676, *r* = 2301*)*	POLAR database
n)	Probability of non-surgical referral if not prescribed opioids or requested imaging first	0.043	*Beta (n* = 36,676, *r* = 1565*)*	POLAR database
	Probability of X-ray if any imaging requested	0.350	*Beta (n* = 22,153, *r* = 7763*)*	Haas R, Gorelik A, O'Connor DA, et al. 2023^17^
	Probability of CT if any imaging requested	0.504	*Beta (n* = 22,153, *r* = 11,160*)*	Haas R, Gorelik A, O'Connor DA, et al. 2023^17^
	Probability of MRI if any imaging requested	0.146		Haas R, Gorelik A, O'Connor DA, et al. 2023^17^
	Probability of surgery if referred to a surgical specialist for LBP	0.0969	*Beta (n* = 70 216, *r* = 243*)*	Jonsson E, Olafsson G, Fritzell P, Hägg O, Borgström F. (2017)^21^
	**Costs**	**AUD**		

a), h)	Cost of an opioid prescription	28.15	*Gamma (sd* = 4.73*)*	Average of cost to the Australian Pharmaceutical Benefits Scheme of a single opioid prescription, weighted by utilisation. (See Supplementary Material)
	Cost of CT scan	230.96		Average benefit paid for MBS item 53,223 (CT of spine) in financial year 21/22
	Cost of X-ray	70.97	*Gamma (sd* = 8.44*)*	Average benefit paid for MBS items 58,106 (lumbosacral x-ray) and 58,109 (sacrococcygeal x-ray), weighted by utilisation in financial year 21/22
	Cost of MRI	367.82	*Gamma (sd* = 38.85*)*	Average of cost to the Australian Medical Benefits Scheme of a single MRI, weighted by utilisation. (See Supplementary Material)
b), g)	Cost of imaging	194.85		Average cost of all imaging types, weighted by probability of each type
c), d), e), f), i), j), k), l), m), n)	Cost of specialist appointment	99.6	*Gamma (sd* = 25.76*)*	Average benefit paid for MBS items 110 and 104, weighted by utilisation, in financial year 21/22
o)	Cost of LBP surgery	27,321	*Gamma (sd* = *16,827)*	Average cost per separation for AR-DRG Version 11 codes I09A, I09B, I09C (spinal fusions), I10A, I10B (other back and neck interventions), B03A, B03B, and B03C (spinal interventions) weighted by utilisation, in financial year 20/21 (inflated to 2022 AUD)

POLAR = Population Level Analysis and Reporting, CT = computed tomography, MRI = Magnetic resonance imaging, LBP = Low Back Pain, AUD = Australian Dollar, MBS = Medical Benefits Scheme, sd = standard deviation, AR-DRG = Australian refined diagnosis-related groups, n = sample size, r = number of occurrences.

The health funder costs attributed to each arm in the decision tree (Table 1) were predominantly derived from national health expenditure data for the Australian Pharmaceutical Benefits Scheme (PBS) and Medical Benefits Scheme (MBS) for the financial year 2021–2022. It is conservatively assumed in these costings that each action represented one opioid prescription, one request for imaging and one referral to a specialist within a year of LBP diagnosis. Utilisation counts and total benefits paid through these federal funding schemes for the item numbers for opioid prescriptions, X-ray, CT or MRI imaging and specialist consultations were used to calculate the mean benefit paid per utilisation (Supplementary Material). To estimate the mean cost of imaging, the mean cost per utilisation calculated for each imaging type was weighted by the proportions of requests for each imaging type.^6^ The cost of surgery was estimated by calculating the mean cost per separation across public hospital separations, thus weighting the cost by utilisation, using eight Australian Refined Diagnosis-Related Group codes for spinal fusions, other back and neck interventions and spinal interventions in 2020/21 (Supplementary Material) (Table 1). These codes were chosen in the base case analysis as they were likely assigned to the majority of surgeries for LBP, but may have underestimated the cost of surgery, as two codes include non-LBP related and probably cheaper treatment. The effect of this was tested in one-way sensitivity analyses.

### Opioid overdose model

**Fig. 2:**
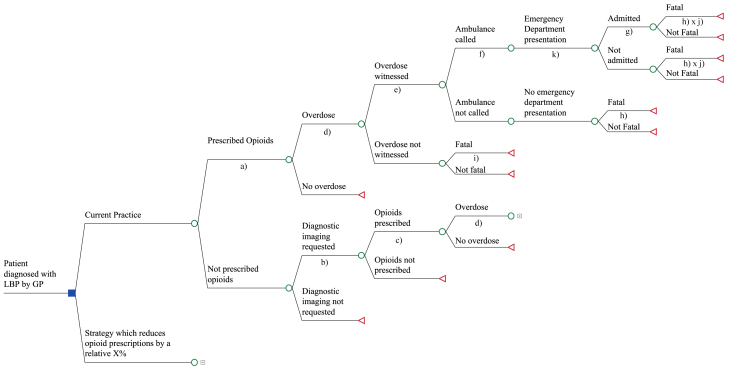
Schematic of opioid harms decision tree model. Square node = decision node. Circle node = chance node. Triangle node = terminal node.

A second decision tree was developed to model opioid overdoses (Fig. 2), with the first several nodes identical to the healthcare actions model, capturing both points at which opioids could be prescribed. After each node at which opioids were prescribed, opioid overdose and downstream acute impacts including overdose-related healthcare use and deaths were modelled.

The model structure was adapted from a recent Australian study modelling the cost-effectiveness of a prescription opioid harm prevention intervention.^22^ In the absence of complete dose information in the POLAR-GP dataset, a low dose of opioids was assumed in all cases. This was a conservative assumption as Neilsen et al.‘s original model incorporated a higher probability of overdose for high doses of opioids. As per the original model, it was also conservatively assumed that a maximum of one opioid overdose could occur per year. The probability of emergency department presentations and hospital admissions following an overdose were also incorporated.

Conditional probabilities in the decision tree (Table 2) were derived from the aforementioned study,^22^ and an additional Australian study.^23^ Costs were derived from a combination of sources as described in Table 2. The cost of opioid prescriptions and imaging were not included in this model to avoid double-counting costs identified in the healthcare actions model.

**Table 2:** Parameters for opioid harms model.

Label	Model parameter	Base estimate	Distribution	Data source
a)	Probability of being prescribed opioids as first action under current practice	0.329	*Beta (n* = 65,612, *r* = 21,603*)*	POLAR database
b)	Probability of having imaging requested if not prescribed opioids as first action	0.167	*Beta (n* = 44,009 *r* = 7333*)*	POLAR database
c)	Probability of having opioids prescribed if not prescribed opioids first, and imaging requested	0.175	*Beta (n* = 7333, *r* = 1280*)*	POLAR database
d)	Probability of overdose if prescribed opioids	0.05		Nielsen S, Scott N, Tidhar T, et al. (2022)^22^
e)	Probability of overdose witnessed	0.490		Nielsen S, Scott N, Tidhar T, et al. (2022)^22^
f)	Probability that ambulance was called	0.400		Nielsen S, Scott N, Tidhar T, et al. (2022)^22^
g)	Probability of admission if attended ED after overdose	0.487	*Beta (n* = *5403, r* = *2629)*	Lam T, Hayman J, Berecki-Gisolf J, et al. (2022)^23^
h)	Proportion who die from witnessed overdose	0.024		Nielsen S, Scott N, Tidhar T, et al. (2022)^22^
i)	Proportion who die from unwitnessed overdose	0.031		Nielsen S, Scott N, Tidhar T, et al. (2022)^22^
j)	Relative risk of death with ambulance	0.883		Nielsen S, Scott N, Tidhar T, et al. (2022)^22^
f)	Cost of ambulance (AUD)	1409.47		Nielsen S, Scott N, Tidhar T, et al. (2022)^22^ (inflated to 2022 AUD)
g)	Cost of opioid hospitalisation (incl ED costs) (AUD)	4250	*Gamma (sd* = *2819)*	Average cost per separation for AR-DRG Version 11 codes V63Z, X62A, X62B, X64A and X64B, weighted by utilisation, in financial year 20/21 (inflated to 2022 AUD)
k)	Cost of ED visit (non-admitted) (AUD)	646		Average cost per non-admitted ED presentation as per the Round 24 NHCDC collected public hospital cost information in financial year 19/20 (inflated to 2022 AUD)^24^

POLAR = Population Level Analysis and Reporting, ED = Emergency Department, AR-DRG = Australian refined diagnosis-related groups, n = sample size r = number of occurrences, AUD = Australian Dollar, NHCDC = National Hospital Cost Data Collection.

### Base case analyses

In the base case analyses, both models were used to estimate the one-year healthcare savings from hypothetical strategies that reduced opioid prescribing by GPs. The cost of implementing a strategy was not incorporated in the model. Healthcare savings were estimated for strategy scenarios resulting in a 0%–100% relative reduction in GP opioid prescribing for LBP, compared to current practice. The relative reduction was assumed to apply to opioids prescribed at any time in the year after the LBP diagnosis. The healthcare savings were estimated as the difference in modelled healthcare costs between the “current practice” and “strategy” arms under each scenario.

To estimate national level savings, the per capita one-year savings were multiplied by the estimate of the number of Australians that present with LBP to a GP, annually (n = 877 700)^25^ (Supplementary Material). The impact of these strategies on opioid overdose deaths was similarly estimated using the opioid overdose model.

### Sensitivity analyses

Probabilistic sensitivity analysis (PSA) was conducted to examine uncertainty around the estimates of annual national healthcare savings and opioid overdose deaths. Using Monte Carlo simulation, 1000 samples were taken from beta distributions for the probabilities and gamma distributions for healthcare costs (Tables 1 and 2) and the sample estimates at the 2.5th and 97.5th percentiles were taken as the 95% uncertainty interval (UI). One-way sensitivity analyses were also conducted to test the potential impact of key model assumptions and parameter uncertainty (Table 3) on healthcare savings and opioid deaths. These analyses were conducted with the opioid reduction strategy set at a 20% relative reduction in prescribing, as this was considered a feasible effect size of an opioid reduction strategy based on previous studies^26^, ^27^, ^28^, ^29^ (Supplementary Material). Results were displayed on tornado plots.

**Table 3:** One-way sensitivity analyses parameters.

Model	Parameter	Base analysis	Sensitivity analyses	Data source
Healthcare actions & opioid harms model	Effect of strategy	Applies to opioid prescribed before and after imaging requests	Applies only to opioids prescribed before imaging requests	NA
Healthcare actions	Probability of surgery if referred to a surgical specialist for LBP	0.0969	±30%	NA
Healthcare actions	Cost of surgery	$27321	$39,493	Average cost per separation for AR-DRG Version 11 codes I09A, I09B, I09C (spinal fusions) weighted by utilisation, in financial year 20/21 (inflated to 2022 AUD) We restricted the set of AR-DRG codes in the sensitivity analysis to ensure only back pain related interventions are used in weighting the costs.
Opioid harms model	Probability of overdose	0.05	0.03–0.10	Nielsen S, Scott N, Tidhar T, et al. (2022)^22^
Opioid harms model	Probability of overdose witnessed	0.49	0.21–0.76	Nielsen S, Scott N, Tidhar T, et al. (2022)^22^
Opioid harms model	Probability that ambulance was called	0.4	0.2–0.8	Nielsen S, Scott N, Tidhar T, et al. (2022)^22^
Opioid harms model (Deaths avoided only)	Proportion who die from unwitnessed overdose	0.031	0.026–0.04	Nielsen S, Scott N, Tidhar T, et al. (2022)^22^
Opioid harms model (Deaths avoided only)	Proportion who die from witnessed overdose	0.024	0.015–0.035	Nielsen S, Scott N, Tidhar T, et al. (2022)^22^

### Effect size for cost-neutral strategy

To estimate the effect size that a hypothetical strategy would need to generate healthcare savings that recoup the costs to implement, i.e. the cost-neutral point, we investigated implementation costs from $0 to $4 million, based on published costs and effects of strategies seeking to influence clinician treatment of musculoskeletal conditions.^26^^,^^30^ For each implementation cost, effect size (relative reduction in opioid prescribing) was varied until the cost-neutral point was attained.

### Role of the funding source

Beyond salary support and funding for software, the funders had no involvement in any aspect of the study.

## Results

### Base case analysis & PSA

**Fig. 3:**
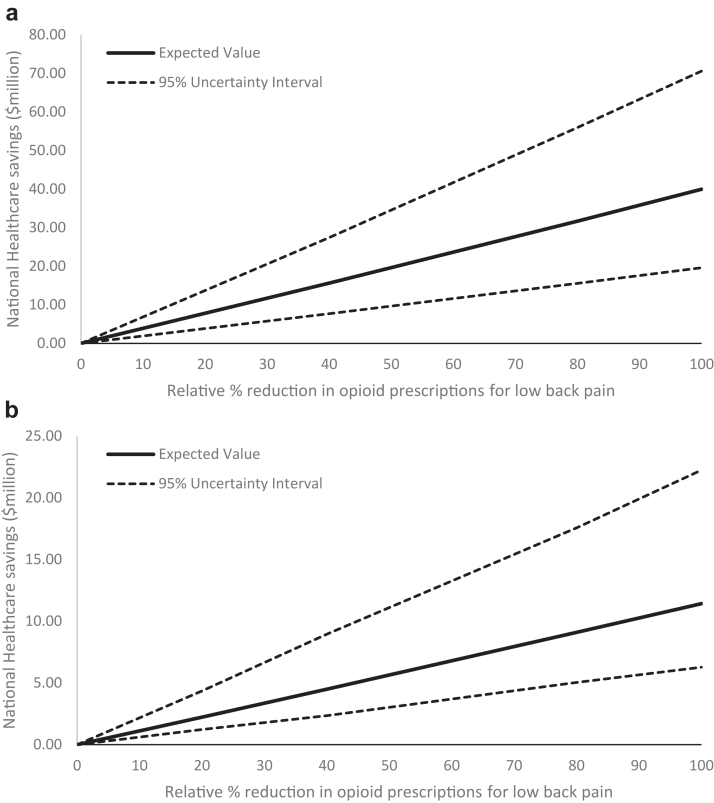
National healthcare savings from strategies that reduce opioid prescriptions from low back pain as predicted from the a) healthcare actions model and b) opioid overdose model.

Anticipated one-year healthcare savings achieved from a strategy that reduces opioid prescribing for LBP patients in general practice by between 0 and 100% are shown in Fig. 3. A 20% relative reduction was estimated to save $6.17 (95% UI: $2.76–$12.81) per LBP patient in a year (through changes in subsequent healthcare) and $2.55 (95% UI $1.40–$4.96) per patient due to avoided opioid overdoses. This equates to savings at a national level of $5.41 million and $2.24 million (total $7.65 million) (Fig. 3a and b). If there was no opioid prescribing at all, the healthcare savings could be as high as $38.50 million.

**Fig. 4:**
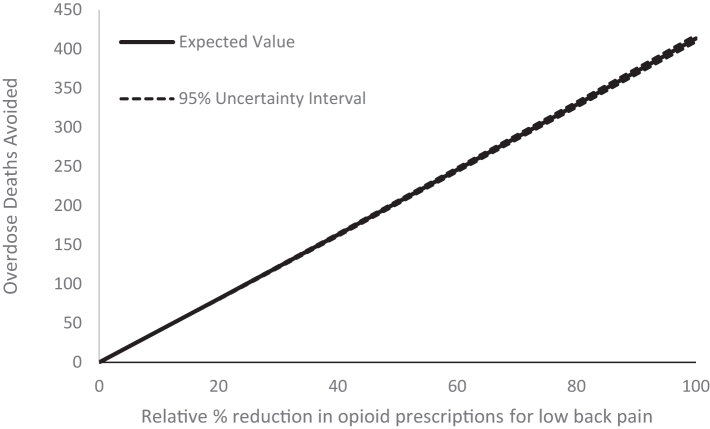
Opioid overdose deaths avoided from strategies that reduce opioid prescriptions from low back pain as predicted from the opioid overdose model.

From the opioid overdose model, a 20% relative reduction in opioid prescribing was predicted to avoid 81 overdose deaths (95% UI 80–82) in a year and up to 414 if no opioids were prescribed at all (Fig. 4).

### One way sensitivity analyses

**Fig. 5:**
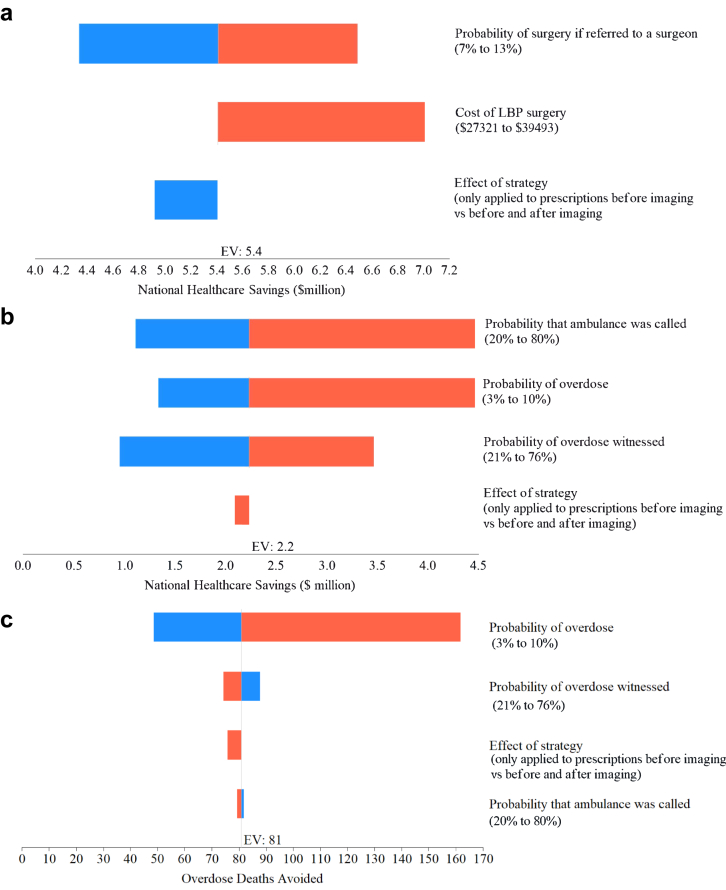
Sensitivity analysis of healthcare savings derived from a) the healthcare actions model, b) the opioid harms model and of c) overdose deaths avoided derived from the opioid harms model.

The one-way sensitivity analyses (Fig. 5) showed that for the healthcare actions model (Fig. 5a), uncertainty in the probability of having surgery following referral to a surgical specialist had the greatest impact on estimates of healthcare savings, while the assumption that the strategy would affect all opioid prescribing vs prescribing as a first action only, had a small impact on estimates. For the opioid overdose model, uncertainty in the probability that an ambulance was called had the greatest impact on estimates of healthcare savings (Fig. 5b), followed by the probability of overdose and the probability that an overdose was witnessed. The predicted overdose deaths were most sensitive to the probability of overdose and was minimally to moderately influenced by uncertainty in the other five parameters tested (Fig. 5c).

### Effect size for cost-neutral strategy

**Fig. 6:**
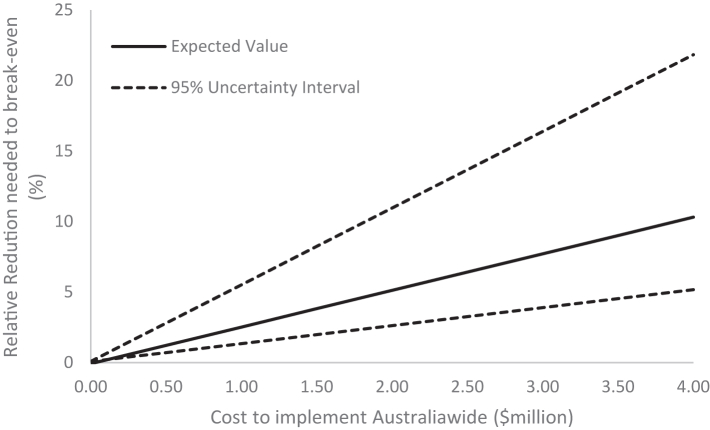
Relationship between cost to implement an intervention Australia-wide and the relative reduction in opioid prescriptions required to ‘break-even’ financially based on the healthcare actions and opioid overdose model combined.

Fig. 6 illustrates the necessary effect size of interventions at different implementation costs, for the strategy to be cost-neutral. A strategy that costs $500,000 to implement, for example, was estimated to require a relative reduction in opioid prescribing of only 1.2% to have these costs recouped in healthcare savings within a year of implementation. A strategy that costs $4 million to implement was estimated to require a 10.3% relative reduction in prescribing to break even.

## Discussion

This study has shown that strategies to reduce opioid prescribing to treat people with LBP in a GP setting have the potential to deliver economic and health benefits, in the first year of implementation. Our modelling has predicted substantial national healthcare savings, based on different levels of opioid prescribing reduction. A strategy that produced a 20% relative reduction in opioid prescribing was estimated to save a total $7.65 million due to changes in downstream care and opioid overdoses avoided (Fig. 3a and b), nationally. We also found that a hypothetical strategy needed relatively small effects on opioid prescribing of up to 10% to recover an investment in a national level program that cost up to $4 million to implement.

For those making decisions about implementing strategies to reduce opioid prescribing, the findings of this study offer an understanding of the short-term economic and health impacts of these decisions. Recent studies on effective strategies to reduce opioid use and prescribing in various contexts have demonstrated effect sizes between 6 and 23% (relative reduction)^26^, ^27^, ^28^, ^29^ (Supplementary Material). Assuming similar reductions could be achieved from strategies to reduce GP prescribing of opioids for LBP at a national level, our models predict healthcare savings from changes to downstream care of $1.6–$6.2 million, and $0.8–$2.6 million in savings from preventing opioid overdoses. More importantly, this could prevent 28–93 overdose deaths within a year of implementation (Fig. 4). Whether net savings are possible will depend on the cost of implementing national level strategies to reduce opioid prescribing.

A recent study on clinician feedback and decision support to reduce imaging in the care of LBP patients trialled in 60% of Australian GPs was estimated to cost a total of $141,154 in 2015 AUD.^30^ If this intervention was upscaled to 100% of Australian GPs, and assuming no economies of scale, it could be expected to cost $267,013 in 2022 AUD. According to our analyses (Fig. 6), an intervention at this cost would only require an effect size of 0.6% (95% UI 0.42%–1.54%) in opioid prescribing to be cost-neutral, which is much lower than the effect size achieved by this intervention on imaging rates (10.9%) and by opioid-focused studies.^26^, ^27^, ^28^, ^29^

This is the first time that the potential healthcare savings associated with reducing opioid prescribing for LBP in primary care have been determined. Our study has a number of strengths. Both models have been presented transparently, have been informed by best available Australian data and incorporate contemporary costs from national healthcare utilisation reports. Where Australian data were not available, appropriate international evidence was applied and inputs were tested in sensitivity analyses. A large administrative database including over 65,000 patients seeking care for LBP was used to develop the healthcare actions model. In previous work, this patient population has been found to be representative of the Australian population receiving LBP care in general practice,^18^ supporting model predictions at a national level.

The study also has a number of limitations. Our models (Figs. 1 and 2) do not incorporate the cost of any opioid prescribing reduction strategies; these costs would need to be subtracted from the savings estimated from our models to calculate the overall cost-saving or cost-effectiveness of a strategy. However, the scenario analyses presented in Fig. 6 do consider the influence of intervention costs. Another limitation is the assumption of causality between the actions in each arm which cannot be confirmed without appropriate trial data. For example, in the POLAR-GP dataset which predominantly informed the structure and probability of events in the healthcare actions model, the prescribing of opioids may have been for a patient's comorbid conditions rather than for LBP. Additionally, the POLAR-GP dataset only includes records of GP actions but does not collect data on patient responses to these: it's possible that patients would not have filled prescriptions, fulfilled imaging requests or visited a specialist from a referral, which may result in an overestimate of some costs and transition probabilities. Also, while the patient population in the POLAR-GP dataset was found to be nationally representative of patients with LBP, it pertained to just one state in Australia (Victoria), and it is possible that GP prescribing in this state differs from prescribing patterns more broadly across Australia. Finally, as with any model, all possible outcomes could not be included and there were potential cost impacts and outcomes associated with reductions in opioid prescribing that could not be accounted for. For example, the savings due to reduced opioid harms other than overdoses and any benefits of the strategy on patients more broadly (other than LBP patients) were not included. Other benefits, such as impacts to the criminal justice system and productivity outcomes, and potential unintended consequences that could incur downstream healthcare costs were also not included.

This study demonstrates that strategies to reduce opioid prescribing to treat LBP have the potential to generate considerable healthcare savings and reduce overdose mortality at a national level within a year of introduction. An understanding of these broad, yet early, population impacts offer those who influence policy and practice decisions a tangible set of outcomes they can expect to be achieved from opioid reduction strategies. Furthermore, the modelling tools developed could facilitate further research in the field. For example, the two models developed could be applied to support evaluations of specific opioid strategies, including cost-effectiveness analysis. An intervention that could be examined in this way could be clinician feedback and decision support for opioid prescribing by GPs, as has been successfully conducted to reduce imaging rates.^30^ Moreover, where relevant input data is available, the models could be adapted to estimate savings from reduced opioid prescribing for other musculoskeletal conditions and to estimate the economic impacts of strategies to reduce imaging for LBP in GP settings. Future research should consider the addition of societal impacts, such as spillover effects on family members, productivity outcomes and impacts to the criminal justice system, and longer-term impacts, so these outcomes can be accounted for in decision-making.

## Contributors

All authors contributed to methodology, data interpretation and writing—review & editing. AK, RH, CGM, RB and AH conceptualised the study. AK, RH and AG derived data inputs for the model. RH, AG and RB had access to the POLAR-GP data as provided by the data custodians and can verify the inputs derived from the dataset. AK conducted all modelled analyses, produced visualisations, wrote the original draft of the manuscript and had final responsibility for the decision to submit for publication.

## Data sharing statement

Some data used in this study are publicly available. Non publicly available data, namely from the POLAR-GP database, are available upon application to the data custodians. The models developed in this study are available upon request to the corresponding author.

## Declaration of interests

AK, RB, CGM, CCL are funded by grants and fellowships from the National Health and Medical Research Council of Australia (APP1171459, APP1194283, APP1194483, APP1193939, APP2006545, APP2015615). RB is additionally funded by the Medical Research Futures Fund, Australian Government, HCF Foundation and Arthritis Australia. She also receives royalties for writing a chapter on Plantar Fasciitis for “UpToDate”. Beyond salary support and funding for software, the funders had no involvement in any aspect of the study.

## References

[1] Global Burden of Disease Low Back Pain Collaborators (2023). Global, regional, and national burden of low back pain, 1990-2020, its attributable risk factors, and projections to 2050: a systematic analysis of the Global Burden of Disease Study 2021. Lancet Rheumatol.

[2] Australian Institute of Health Welfare (2023).

[3] Australian Commission on Safety and Quality in Health Care (2022). https://www.safetyandquality.gov.au/sites/default/files/2022-08/low_back_pain_clinical_care_standard.pdf.

[4] Foster N.E., Anema J.R., Cherkin D. (2018). Prevention and treatment of low back pain: evidence, challenges, and promising directions. Lancet.

[5] Kamper S.J., Logan G., Copsey B. (2020). What is usual care for low back pain? A systematic review of health care provided to patients with low back pain in family practice and emergency departments. Pain.

[6] Haas R., Gorelik A., O’Connor D.A., Pearce C., Mazza D., Buchbinder R. (2023). Patterns of imaging requests by general practitioners for people with musculoskeletal complaints: an analysis from a primary care database. Arthritis Care Res (Hoboken).

[7] Williams C.M., Maher C.G., Hancock M.J. (2010). Low back pain and best practice care: a survey of general practice physicians. Arch Intern Med.

[8] Haas R., Gorelik A., O'Connor D. (2023). 44 Opioid prescription patterns in general practice for people with regional musculoskeletal pain: a longitudinal primary care database study. BMJ Evid Based Med.

[9] Jones C.M.P., Day R.O., Koes B.W. (2023). Opioid analgesia for acute low back pain and neck pain (the OPAL trial): a randomised placebo-controlled trial. Lancet.

[10] Webster B.S., Verma S.K., Gatchel R.J. (2007). Relationship between early opioid prescribing for acute occupational low back pain and disability duration, medical costs, subsequent surgery and late opioid Use. Spine (Philadelphia, Pa 1976).

[11] Chou R., Turner J.A., Devine E.B. (2015). The effectiveness and risks of long-term opioid therapy for chronic pain: a systematic review for a National Institutes of Health Pathways to Prevention Workshop. Ann Intern Med.

[12] Fritz J.M., Brennan G.P., Hunter S.J., Magel J.S. (2013). Initial management decisions after a new consultation for low back pain: implications of the usage of physical therapy for subsequent health care costs and utilization. Arch Phys Med Rehabil.

[13] Buchbinder R., van Tulder M., Öberg B. (2018). Low back pain: a call for action. Lancet.

[14] Eccleston C., Eccleston C., Fisher E. (2017). Interventions for the reduction of prescribed opioid use in chronic non-cancer pain. Cochrane Database Syst Rev.

[15] Alperovitch-Najenson D., Becker A., Belton J. (2023).

[16] Australian Institute of Health and Welfare (2023).

[17] Haas R., Busija L., Gorelik A. (2021). Patterns of care for people presenting to Australian general practice with musculoskeletal complaints based on routinely collected data: protocol for an observational cohort study using the Population Level Analysis and Reporting (POLAR) database. BMJ Open.

[18] Haas R., Gorelik A., Busija L. (2023). Prevalence and characteristics of musculoskeletal complaints in primary care: an analysis from the population level and analysis reporting (POLAR) database. BMC Primary Care.

[19] Department of Health and Aged Care (2021). Victoria primary health networks (PHN) resource collection. https://www.health.gov.au/resources/collections/victoria-primary-health-networks-phn-resource-collection.

[20] Australian Bureau of Statistics (2024). https://dbr.abs.gov.au/region.html?lyr=aus&rgn=AUS.

[21] Jonsson E., Olafsson G., Fritzell P., Hägg O., Borgström F. (2017). A profile of low back pain: treatment and costs associated with patients referred to orthopedic specialists in Sweden. Spine (Philadelphia, Pa 1976).

[22] Nielsen S., Scott N., Tidhar T., Quiroga M.D.M., Lenton S., Dietze P. (2022). The cost and impact of distributing naloxone to people who are prescribed opioids to prevent opioid-related deaths: findings from a modelling study. Addiction.

[23] Lam T., Hayman J., Berecki-Gisolf J., Sanfilippo P., Lubman D.I., Nielsen S. (2022). Pharmaceutical opioid poisonings in Victoria, Australia: rates and characteristics of a decade of emergency department presentations among nine pharmaceutical opioids. Addiction.

[24] Independent Hospital Pricing Authority (2022).

[25] NPS MedicineWise (2020).

[26] Coombs D.M., Machado G.C., Richards B. (2021). Effectiveness of a multifaceted intervention to improve emergency department care of low back pain: a stepped-wedge, cluster-randomised trial. BMJ Qual Saf.

[27] Liebschutz J.M., Xuan Z., Shanahan C.W. (2017). Improving adherence to long-term opioid therapy guidelines to reduce opioid misuse in primary care: a cluster-randomized clinical trial. JAMA Intern Med.

[28] Sandhu H.K., Booth K., Furlan A.D. (2023). Reducing opioid use for chronic pain with a group-based intervention: a randomized clinical trial. JAMA.

[29] Gupta A., Lindstrom S., Shevatekar G. (2020). Reducing opioid overprescribing by educating, monitoring and collaborating with clinicians: a quality improvement study. Cureus.

[30] Morgan T., Wu J., Ovchinikova L., Lindner R., Blogg S., Moorin R. (2019). A national intervention to reduce imaging for low back pain by general practitioners: a retrospective economic program evaluation using Medicare Benefits Schedule data. BMC Health Serv Res.

