# Supplementary material for: Cost savings of reducing opioid prescribing for the treatment of people with low back pain in general practice: a modelling study
Source: Lancet Reg Health West Pac. 2025 Jan 10;54:101277. doi: 10.1016/j.lanwpc.2024.101277 (PMC11773042; doi:10.1016/j.lanwpc.2024.101277)
Supplement: Supplementary Material [file mmc1.pdf]

Supplementary Materials

Contents

Estimating the mean cost of an opioid prescription 2

Estimating the mean cost of imaging 3

Estimating the mean cost of surgery 4

Estimating the number of patients visiting a general practitioner for low back pain 5

Calculation of relative reduction achieved in previous studies 6

CHEERS 2022 Checklist 7

Estimating the mean cost of an opioid prescription

To estimate the mean cost of an opioid prescription, we first downloaded the utilisation (services) and benefit reports for July 2021 to June 2022, for PBS item numbers related to any opioid, from the Australian Government, Medicare Statistics website: http://medicarestatistics.humanservices.gov.au/statistics/pbs_item.jsp

The reports for the following item numbers were downloaded: 8865N, 8866P, 8867Q, 10746N, 10755C, 10756D, 10770W, 1214X, 5063L, 12054K, 12065B, 1215Y, 3316M, 12022R, 12066C, 5265D, 5277R, 5278T, 5279W, 5280X, 5437E, 5438F, 5439G, 5440H, 5441J, 8878G, 8891Y, 8892B, 8893C, 8894D, 5116G, 8420E, 8421F, 8541M, 8542N, 8543P, 9299K, 9406C, 9407D, 9408E, 9409F, 12016K, 12046B, 12047C, 1606M, 1609Q, 1644M, 1645N, 1646P, 1647Q, 2122Q, 2123R, 2124T, 5168B, 10864T, 10869C, 10874H, 10878M, 12009C, 1653B, 1654C, 1655D, 1656E, 2839K, 2840L, 2841M, 5237P, 5238Q, 8035X, 8146R, 8305D, 8306E, 8349K, 8489T, 8490W, 8491X, 8492Y, 8493B, 8494C, 2481N, 2622B, 5190E, 5191F, 5195K, 5197M, 8385H, 8464L, 8501K, 8502L, 8644Y, 9399Q, 12023T, 12031F, 12044X, 12048D, 12074L, 8386J, 8387K, 8388L, 9400R, 8000C, 8934F, 8935G, 8936H, 10757E, 10758F, 10776E, 11102H, 11111T, 10091D, 10092E, 10094G, 10096J, 10100N, 2527B, 5232J, 8455B, 8523N, 8524P, 8525Q, 8582Q, 8843K, 12008B, 12024W

The mean benefit per utilisation of all item numbers was calculated as sum of all benefits/ sum of count of services. The standard deviation, which was used for probabilistic sensitivity analysis, was calculated from the mean cost per utilisation of each class of opioids.

Estimating the mean cost of imaging

To estimate the mean cost of an imaging service, the mean cost for CT scans, X-rays and MRIs were first calculated separately. The utilisation (services) and benefit reports from July 2021 to June 2022, for MBS item numbers related to each of these services were first downloaded from the Australian Government, Medicare Statistics website:

http://medicarestatistics.humanservices.gov.au/statistics/mbs_item.jsp

For CT scans, the item reports for MBS Item 53223 were downloaded.

For X-rays, the item reports for MBS Item numbers 58106 and 58109 were downloaded.

For MRIs, the item reports for MBS Item numbers 63111, 63114, 63125, 63128, 63131, 63151, 63154, 63161, 63164, 63167, 63170, 63173, 63176, 63179, 63182, 63185, 63201, 63204, 63219, 63222, 63225, 63228, 63231, 63234, 63237, 63240, 63243, 63271, 63274, 63277, 63280, 63510, 63554, and 63557 were downloaded.

The mean cost per utilisation and standard deviation for this cost for each type of imaging service was calculated from the number of services and benefits reported for each item number.

The mean cost of imaging was calculated within the Treeage software used for the modelling, using the following formula:

$\text{Weighting}_{CT}\text{xMean Cost}_{CT} + \text{Weighting}_{X\text{-}ray}\text{xMean Cost}_{X\text{-}ray} + \text{Weighting}_{MRI}\text{xMean Cost}_{MRI}$

The weightings were derived from the following study: Haas R, Gorelik A, O'Connor DA, Pearce C, Mazza D, Buchbinder R. Patterns of Imaging Requests By General Practitioners for People With Musculoskeletal Complaints: An Analysis From a Primary Care Database. Arthritis Care Res (Hoboken). 2023; https://doi.org/10.1002/acr.25189

Weighting_{CT} = 11160/22153 = 0.504, Mean Cost_{CT} = 230.96

$\text{Weighting}_{X\text{-}ray}$ = 7763/22153 = 0.350, $\text{Mean Cost}_{X\text{-}Ray}$ = 70.97

$\text{Weighting}_{MRI\,=}$ 3230/22153 = 0.146, Mean Cost_{MRI} = 367.86

Within Treeage, standard deviations for the mean cost for each type of imaging service and the numerator and denominators used to calculate the weightings were used to define distributions from which to sample from in probabilistic sensitivity analysis.

Estimating the mean cost of surgery

The mean cost of surgery was estimated using data from the following government document:

Independent Hospital and Aged Care Pricing Authority. National Hospital Cost Data Collection (NHCDC) Public Sector Report 2020-21. Darlinghurst 2023.

The downloadable excel document labelled "NHCDC Public Sector Cost Weights AR-DRG Version 11.0 2020-21" reports cost weights, total costs and number of separations for each Australian refined diagnosis-related group (AR-DRG) in the financial year 2020-21. Each AR-DRG relates to a code applied for each hospitalisation, depending on the characteristics of that hospitalisation, including procedures conducted. The primary use of these codes is for activity-based funding of public hospitals.

In this study, AR-DRG codes for hospitalisations that included relevant surgeries were used to estimate the mean cost of a surgery. In the base case analysis, the following AR-DRG codes were used: I09A, I09B, I09C, I10A, I10B, B03A, B03B, B03C which related hospitalisations that included "Spinal Fusions", "Other back and neck interventions" and "Spinal interventions".

The mean cost per surgery was calculated as sum of the total costs/ sum number of separations for each AR-DRG code. The standard deviation, which was used for probabilistic sensitivity analysis, was calculated from the mean cost per separation of each AR-DRG code.

In one-way sensitivity analysis, smaller group of AR-DRG codes (I09A, I09B, and I09C), related only to spinal fusions, was used to calculate the mean cost per surgery.

Estimating the number of patients visiting a general practitioner for low back pain

To calculate national level healthcare savings in this study, an estimate of the number of Australians that present with LBP to a GP in a year was needed. Information used in the following document was used for this estimate:

NPS MedicineWise. General Practice Insights Report July 2018–June 2019. Sydney; 2020.

This document reported that a total of 21,942,493 patients visited a GP in 2018-19 and that 4% of patients recorded a low back pain condition within the year. Therefore, we estimated that the number of Australians presenting to a GP with LBP to be 0.04 x 21,942,493 = 877700.

Calculation of relative reduction achieved in previous studies

In the discussion of the main manuscript, use the findings of previous studies assessing the effect of strategies to reduce opioids and influence clinician behaviour to put our findings into context. Most of these studies do not directly report relative reductions in outcomes, so we calculated these relative reductions from the results presented in the papers. These calculations are presented below.

Coombs DM, Machado GC, Richards B, Needs C, Buchbinder R, Harris IA, et al. Effectiveness of a multifaceted intervention to improve emergency department care of low back pain: a stepped-wedge, cluster-randomised trial. BMJ Qual Saf. 2021;30(10):825-35.

At the end of the trial reported in this study, 50.5% of the intervention group received any opioid medications from a clinician, while 62.8% of the control group received any opioid medication. Therefore, the intervention resulted in a 62.8-50.5 = 12.3% absolute reduction in opioids received, which equates to a 12.3/62.8 = **19.6%** relative reduction in opioids received.

Liebschutz JM, Xuan Z, Shanahan CW, LaRochelle M, Keosaian J, Beers D, et al. Improving Adherence to Long-term Opioid Therapy Guidelines to Reduce Opioid Misuse in Primary Care: A Cluster-Randomized Clinical Trial. JAMA Intern Med. 2017;177(9):1265-72.

At the end of the trial reported in this study, 21.3% of the intervention group discontinued opioid prescriptions and 16.8% in the control group discontinued opioid prescrptions. Therefore, the intervention resulted in a 21.3-16.8 = 4.5% absolute reduction continued opioid prescriptions which equates to 4.5/(100-16.8) = **5.6%** relative reduction in continued opioid prescriptions.

Sandhu HK, Booth K, Furlan AD, Shaw J, Carnes D, Taylor SJC, et al. Reducing Opioid Use for Chronic Pain With a Group-Based Intervention: A Randomized Clinical Trial. JAMA. 2023;329(20):1745-56.

At the end of the trial reported in this study, 29% of the intervention group discontinued opioid use and 7% of the usual care group discontinued opioid use. Therefore, the intervention resulted in s 29-7 = 22% absolute reduction in opioid use, which equates to a 22/(100-7)=**23%** relative reduction in continued opioid use.

Gupta A, Lindstrom S, Shevatekar G. Reducing Opioid Overprescribing by Educating, Monitoring and Collaborating with Clinicians: A Quality Improvement Study. Cureus. 2020;12(4):e7778.

This was an interupted time series analysis in which a **7%** relative risk reduction in schedule II opiate orders after the intervention was directly reported.

CHEERS 2022 Checklist

Topic	No.	Item	Location where item is reported
**Title**			
	1	Identify the study as an economic evaluation and specify the interventions being compared.	NA - study is a modelling study that reports on healthcare costs and mortality but is not a full economic evaluation.
**Abstract**			
	2	Provide a structured summary that highlights context, key methods, results, and alternative analyses.	Page 3
**Introduction**			
**Background and objectives**	3	Give the context for the study, the study question, and its practical relevance for decision making in policy or practice.	Page 4
**Methods**			
**Health economic analysis plan**	4	Indicate whether a health economic analysis plan was developed and where available.	NA- separate plan not developed, but is described within the manuscript.
**Study population**	5	Describe characteristics of the study population (such as age range, demographics, socioeconomic, or clinical characteristics).	Page 5
**Setting and location**	6	Provide relevant contextual information that may influence findings.	Page 5-6
**Comparators**	7	Describe the interventions or strategies being compared and why chosen.	Page 6-7
**Perspective**	8	State the perspective(s) adopted by the study and why chosen.	Page 5
**Time horizon**	9	State the time horizon for the study and why appropriate.	Page 5
**Discount rate**	10	Report the discount rate(s) and reason chosen.	Page 5
**Selection of outcomes**	11	Describe what outcomes were used as the measure(s) of benefit(s) and harm(s).	Page 6-7
**Measurement of outcomes**	12	Describe how outcomes used to capture benefit(s) and harm(s) were measured.	Page 6 (see 'deaths' as outcome)
**Valuation of outcomes**	13	Describe the population and methods used to measure and value outcomes.	Page 6
**Measurement and valuation of resources and costs**	14	Describe how costs were valued.	Page 5-6
**Currency, price date, and conversion**	15	Report the dates of the estimated resource quantities and unit costs, plus the currency and year of conversion.	Page 5-6

Topic	No.	Item	Location where item is reported
**Rationale and description of model**	16	If modelling is used, describe in detail and why used. Report if the model is publicly available and where it can be accessed.	Pages 4-7
**Analytics and assumptions**	17	Describe any methods for analysing or statistically transforming data, any extrapolation methods, and approaches for validating any model used.	Page 4-7, 9
**Characterising heterogeneity**	18	Describe any methods used for estimating how the results of the study vary for subgroups.	NA
**Characterising distributional effects**	19	Describe how impacts are distributed across different individuals or adjustments made to reflect priority populations.	NA
**Characterising uncertainty**	20	Describe methods to characterise any sources of uncertainty in the analysis.	Page 7
**Approach to engagement with patients and others affected by the study**	21	Describe any approaches to engage patients or service recipients, the general public, communities, or stakeholders (such as clinicians or payers) in the design of the study.	Page 10
**Results**			
**Study parameters**	22	Report all analytic inputs (such as values, ranges, references) including uncertainty or distributional assumptions.	Tables 1-3
**Summary of main results**	23	Report the mean values for the main categories of costs and outcomes of interest and summarise them in the most appropriate overall measure.	Page 7, Figures 3-4, 6
**Effect of uncertainty**	24	Describe how uncertainty about analytic judgments, inputs, or projections affect findings. Report the effect of choice of discount rate and time horizon, if applicable.	Page 7-8, Figure 3-6
**Effect of engagement with patients and others affected by the study**	25	Report on any difference patient/service recipient, general public, community, or stakeholder involvement made to the approach or findings of the study	Page 10 - advice provided at early stages of study to determine aims of the modelling
**Discussion**			
**Study findings, limitations, generalisability, and current knowledge**	26	Report key findings, limitations, ethical or equity considerations not captured, and how these could affect patients, policy, or practice.	Page 8-10
**Other relevant information**			
**Source of funding**	27	Describe how the study was funded and any role of the funder in the identification, design, conduct, and reporting of the analysis	Page 10
**Conflicts of interest**	28	Report authors conflicts of interest according to journal or International Committee of Medical Journal Editors requirements.	Page 10 and separate forms

From: Husereau D, Drummond M, Augustovski F, et al. Consolidated Health Economic Evaluation Reporting Standards 2022 (CHEERS 2022) Explanation and Elaboration: A Report of the ISPOR CHEERS II Good Practices Task Force. Value Health 2022;25. doi:10.1016/j.jval.2021.10.008
